# Hospital Sunday in London

**Published:** 1895-06-22

**Authors:** 


### The Institutional Workshop.

### HOSPITAL SUNDAY IN LONDON.

Motto for the day: "He gives twice, who makes his neighbour give also."

### A BRIEF HISTORY.

To the late Dr. James Wakley belongs the credit of having inspired others to combine in a special effort to raise the amount collected on Hospital Sunday in London to a sum equal to the average annual deficiency of all the participating hospitals. In 1873, the year this Fund was instituted, only 1,072 congregations contributed, and the total receipts amounted to £27,700. There was an increase of £2,000 in the amount collected in 1874, and then the total receipts gradually diminished till they reached the minimum of £24,905 in 1878. An increase was then perceptible year by year, till the receipts rose to £39,330 in 1884, when the contributing congregations amounted to 1,522. In 1885 the total receipts fell to £34,320, and in that year Dr. James Wakley, who was suffering from a mortal disease, sent for the writer and urged him to devise some scheme which would help the hospitals by increasing the interest in and collections on Hospital Sunday. After due deliberation, a number of public meetings were organised throughout London, at which some of the most eminent leaders of public opinion kindly consented to speak. Dr. Wakley, as editor of the *Lancet*, published a special supplement, which was edited by Mr. Burdett, and widely circulated. In the result, the total receipts in 1886, although the congregations only amounted to 1,595, reached £40,399. With a view of still further stimulating the interest of the public in the voluntary system of hospital support, Dr. Wakley and the present writer discussed together the outlines of a newspaper which should deal with all the questions involved by the maintenance of an adequate hospital system. In the result, the first number of The Hospital was published on October 2nd, 1886. In the year 1887 the Council of the Metropolitan Hospital Sunday Fund continued the system of holding meetings in the week preceding Hospital Sunday. These meetings entailed a good deal of organisation, expense, and trouble, and the number held has gradually diminished until 1894, when the Lord Mayor, in co-operation with Sir Sydney Waterlow and Mr. Burdett, gave a conversazione, to which he invited all the preachers on Hospital Sunday.

In the year (1887) following Dr. Wakley's death, the proprietors of the *Lancet* discontinued the issue of special supplements, and The Hospital filled the void thus created, by publishing a special supplement that year, and has continued the practice ever since. In 1888 the *Lancet* again issued a special supplement, and the present proprietors, like The Hospital, continued to do so, and have circulated many thousands of abstracts from it amongst the congregations each year. Up to, and including the year 1892, the total receipts always exceeded £40,000, the maximum being reached in 1891, when they amounted to £45,331, including donations of £5,000 from the late Duke of Cleveland, and £1,000 from Sir Savile Crossley, Bart., the latter of whom has since given a similar sum annually. In 1893 the total receipts fell to £39,291, and in 1894 to £38,680, exclusive of a legacy of £5,000 bequeathed by the late Mr. W. I. Whitaker. For nearly twenty years Mr. Henry N. Custance, the present secretary of the Fund, has laboured assiduously and successfully to increase the number of contributing congregations, which now amount to about 1,800, as compared with the 1,072 at the outset. By his attention to the requirements of the poor who seek hospital and convalescent aid, and surgical appliances through the agency of the Fund, Mr. Custance has rendered yeoman's service. Still, the fact that the amounts received from congregations fell to £35,962 in 1894, the smallest sum received, with one exception, in any year since 1886, induced the proprietors of The Hospital to organise a special effort during the present year, in the hope that the total receipts on Hospital Sunday may be lifted out of the rut of £40,000, and increased to £60,000 or £70,000, the latter being the actual sum needed to place the hospitals in funds.

### What Has Been Done in 1895.

After a good deal of thought, we determined to publish a special supplement, containing diagrams and illustrations which should bring to the mind of everybody the actual circumstances of the hospitals, the work which they do for every branch of the community, and their financial position. A first edition of 20,000 copies was printed and circulated amongst the preachers and congregations throughout London. In addition to this, the proprietors of The Hospital, in conjunction with seven other gentlemen, provided a special fund, amounting to some hundreds of pounds, which was devoted to the circulation of the special supplement amongst the well-to-do residents in every part of the metropolis. These special supplements were circulated, as far as possible, by special messenger, or by post, accompanied by a letter, and a subscription form addressed to the Lord Mayor. The co-operation of the editors of the leading daily, evening, and weekly papers was invited, and, in the result, an amount of space and attention has been attracted to the hospital question in London, which has never been equalled in the history of the Fund. Our thanks are specially due to the editors and proprietors of the *Times*, the *Daily News*, the *Morning Post*, *Daily Chronicle*, *Standard*, *Daily Graphic*, *Morning Advertiser*, the *Morning*, the *Globe*, *Pall Mall Gazette*, *St. James's Gazette*, *Westminster Review*, *Financial Times*, *Financial News*, *City Press*, *Observer*, *Weekly Sun*, *Christian World*, *Church Times*, and many other papers. Everybody seems to have felt that, as citizens of a great city, it was a privilege to help in so good a cause as that represented by the voluntary hospitals. It is interesting and remarkable that the proprietors of the *Times* should have consented to publish the diagrams illustrating the proportion of the revenue of the hospitals contributed by the living, the dead hand, and the patients themselves. In the result, it is satisfactory to know that no one resident in London who is really able to subscribe liberally to our hospitals can have failed to have had the question brought fully under his or her personal notice during the week. If the effort to raise the sum of £70,000 fail, the responsibility will not rest with the Press or the clergy and ministers of religion, but must lie with the individual residents in London themselves, who may fail to avail themselves of the high privilege of taking part in the noble work of helping the sick to recover their health and become useful members of society. We feel that our thanks are due to the whole of our colleagues in the Press, as well as to the hundreds of preachers who advocated the cause of the hospitals from the pulpits of London last Sunday. There was hardly a pulpit from which the facts and statements contained in our special supplement were not brought to the notice of the congregations with forcible and convincing eloquence. From the Archbishop of Canterbury to the humblest missioner in the smallest conventicle, where collections were made on behalf of the hospitals, everybody seems to have put his whole heart into the work of arousing Londoners to their responsibilities in regard to this question. London is a vast city, the population of which it is difficult to arouse to a feeling of responsibility on a question like this. Still, Hospital Sunday, 1895, will be memorable from the circumstance that everywhere, through the press and from the pulpit, the most strenuous effort has been made to carry conviction into the minds, and to arouse the sympathies of every class of the community on behalf of the hospitals.

### The Position of Non-Givers.

The primary difficulty is to induce the vast majority of the inhabitants of London to give anything substantial to the hospitals at all, or indeed to anything. It was noticeable, for instance, that the occupants of some of the best seats at Westminster Abbey at the morning service were ladies, wearing expensive dresses, who responded to the eloquent appeal of the Dean by contributing one penny each. This matter was brought to our personal knowledge, and an examination of the actual coins given revealed the fact that the majority of the congregation failed to realise their responsibility, while a few of them were so callous as to give nothing—or worse than nothing, for they contributed foreign coins of no value. Complaints have reached us from other sources because the special effort made this year—thanks to the Press—has brought the subject of Hospital Sunday during the past week under the immediate notice of thousands of people who take no interest in the movement whatever. This class of person urges that they are "sick to death" of hearing of Hospital Sunday, and that it is a nuisance to have so much fuss made about the hospitals, their needs and work, which are no concern of theirs. Can any man or woman who gives expression to such opinions as these realise their individual responsibility in the matter? We are convinced that they do not; but one day, when suffering from illness or accident, they may perchance remember these neglected opportunities of taking their part in the noblest of all charities, and regret that they did not act the Good Samaritan when the opportunity offered, as it does offer to every resident in London, on Hospital Sunday. With the object of securing that the special supplement and letter should reach the hands of those for whom it was intended, the envelopes were marked, "Important; by special messenger." We have reason to know that this plan has succeeded in placing the facts before every well-to-do resident, but this success has called forth a protest from one gentleman whose name has been much in the papers during the week, and who protests against the plan pursued. "Fortunately," he writes, "I was at home, therefore no trouble or expense was incurred, but had I been a hundred miles away, this *unimportant* communication would have been sent on to me." Of course it would have been sent on to him. That was why the missive was dispatched, and the least he can do is to forward a sum to the Lord Mayor which will be equal to the expense which he implies would have been incurred by sending the appeal a hundred miles by special messenger. At any rate, the receipt of our special supplement has brought the needs of the hospitals to his notice, and it must rest with him to determine whether he will do his duty by contributing to the hospitals, or whether he will be content to incur the grievous responsibility of refusing to minister to the needs of the sick poor in the great city where he habitually dwells. These instances, and we might quote others, go to prove the indifference of thousands of the community to the circumstances of their poorer neighbours, and reveal a state of feeling which is appalling in its total disregard of the privileges and duties of citizenship of a great city like London. In the epistle for the day, St. John says, "Who that loveth not his brother whom he hath seen, how can he love God, whom he hath not seen." How, indeed! And in the Gospel taken from St. Luke, at the end of the parable of Dives and Lazarus, we have it further recorded, "If they hear not Moses and the prophets, neither will they be persuaded though one rose from the dead." Our feeling and belief is that the case of non-givers is so terrible in its responsibility that it should excite our commiseration and pity, because they voluntarily deprive themselves of the greatest pleasure which mortal men can experience, the privilege of appreciating the value of money by ministering to the necessities of those less fortunate than themselves. We hope that the eloquent words heard on Hospital Sunday, and read by Londoners during the week, in relation to hospitals, may induce thousands of people to endeavour to carry out our motto for Hospital Sunday: "He gives twice who makes his neighbour give also."

### The Sick Child and the Bishop of London.

A hospital is a many-sided institution, full of suffering, sadness, joy, hope, pathos, reality, life, and death. It happened not long ago that a poor boy of tender years was stricken by a mortal disease. He had been a great help to his parents, and through the kindness of the clergyman in the parish where they resided, he found his way into one of the great London hospitals. There he was visited by the clergy of the parish. It became evident that the child could not recover, and he manifested a knowledge of God and an assured certainty of his future life which was most remarkable. Amongst other things, he desired to have the Holy Communion administered to him, but nothing would satisfy the child, except that he should be confirmed before he received the Sacrament. The clergy were in a difficulty, and in the end they laid the case before the Bishop of London. He was coming to a confirmation service at a church some distance from the hospital, and promised to come on there and confirm the little boy afterwards. When the Bishop arrived the child was wheeled into the hospital chapel, propped up in a chair, which made his terrible weakness all the more apparent. The boy's face was irradiated with joy at the fulfilment of his wish, and no one who was present will ever forget that confirmation, which moved the hearts of all, and caused the Bishop to shed tears. After the service the boy was wheeled back into the ward, and the Bishop of London was so interested that he followed the child there, and helped to place him in his cot. There were a few others present who were engrossed in this child, and whilst the attention of all of them was absorbed in the little boy who had just been confirmed, a deep sob was heard by all present. The Bishop turned round and saw a dirty, ill-clad woman leaning over a cot where another child lay a-dying. In an instant the Bishop took in the scene, the distress of this poor mother at the thought that her little one was passing away unnoticed when so much was being done for the other little sufferer. It touched him deeply, and he took the woman in his arms and comforted her, and made much of her, and the mother's face changed, and her tears were dried, and there were two happy death-beds in that ward that night. Would that we could persuade the indifferent, the pleasure seeker, the people who find life so dull, to visit some hospital which may be near the place where they live, at any rate once or twice a year. What a change it would make in their lives; how happy they might become, and how soon then would it be unnecessary for press men and preachers to urge with all the force of conviction and power of eloquence the privilege of supplying the hospitals with all the funds that they need to adequately meet the wants of the sick poor of London.

A Word to the Secretaries.

We are immensely indebted to the officers of hospitals everywhere, and especially to the secretaries. It would delight us to be able to cause contributions to flow in due proportion into the letter-box of every hospital secretary who needs additional funds for the support of the institution for the maintenance of which he is mainly responsible, The special effort made this year may, or may not, produce the desired result. It has, at any rate, awakened an interest in the metropolitan hospitals and in hospitals generally, which has probably been unequalled for many years past. Years ago, under somewhat similar circumstances, an astute secretary, at our suggestion, issued one thousand appeals so soon as the Hospital Sunday collections had been made, and in the result he received £1,000. Here is a word to the wise, which we hope may be acted upon without delay, and with a result that may bring joy to the heart of every zealous secretary throughout London.

